# Threshold-distance functions predict speech recognition with cochlear implants

**DOI:** 10.1038/s41598-025-29475-4

**Published:** 2025-11-28

**Authors:** E. Kludt, S. Ewald, N. Prenzler, R. Salcher, K. Willenborg, M. Sato, A. Büchner, A. Kral, T. Lenarz

**Affiliations:** 1 Clinics of Otolaryngology, Head and Neck Surgery, Hannover Medical School, Hannover, Germany; 2 Hearing4all Cluster of Excellence, Hannover Medical School, Hannover, Germany

**Keywords:** Cochlear health, Auditory nerve degeneration, Compound action potentials, Pull-back technique, Neuronal survival, Neuroscience, Medical research

## Abstract

Cochlear implantation shows unexplained outcome variability. Among the key factors affecting outcomes measured by speech understanding is the functional state of the auditory nerve and the amount of its degeneration due to hearing loss. In the present study we actively varied the distance of the stimulating electrodes from the modiolus (the spiral ganglion), quantified it using fluoroscopy and cone-beam computed tomography and related it to electrically-evoked compound action potentials (eCAPs) in human subjects. Stimulation was monopolar. The distance from modiolus could explain up to 91% of the variability in the thresholds of eCAPs. The threshold varied between participants. For individual electrodes of the implant in the given participant, a linear relationship between threshold (in current level) and distance from modiolar axis was found, with different slopes in different participants. The slopes of the eCAP thresholds to modiolus distance of the electrode across ears could explain up to 67% of the variability of speech understanding in these participants. We suggest that the slope of the threshold-distance function might serve as a marker for the functional state of the target neurons (spiral ganglion cells) and can be used to assess this in individual subjects.

## Introduction

Cochlear implants (CIs) represent a highly effective compensation of severe to profound hearing loss, capable of providing excellent speech recognition. However, speech outcomes vary significantly between different patients. Such outcome variability remains an unresolved issue in cochlear implantation^1,2^. The factors contributing to outcome variability are in general known and include peripheral, central and device-related factors (adults^3–8^, children^4,9–11^). In a given individual, however, there is limited knowledge which factor contributed to the performance and which is the limiting one. Therefore, considerable amount of the outcome variability in individual cochlear-implanted patients remain unexplained^11–13^. While best performance in monosyllabic tests can exceed 90% in some (particularly pediatric) patients^14^, the mean performance remains around 60% in most cohorts studied^5^. Exploring the factors contributing to “underperformance” of some subjects^2^ remains a challenge.

One potential factor in this variability is the condition of the cochlear nerve (so-called “cochlear health” or “neural health”^15,16^) that may determine how well information can be transmitted from the CI to the central auditory system. Neural health comprises several factors, including the overall survival and dystrophic changes of the spiral ganglion cells in the course of persistent deafness, the extent of the degeneration of primary afferents, but also the general responsiveness to electric stimulation. It is currently challenging to assess the neural health in an individual subject.

Few functional biomarkers of neural health in CI patients have been proposed^17–19^. Electrically-evoked compound action potentials provide a way to assess the responsiveness of the auditory nerve^20^. A combined acoustic response of hair cells and spiral ganglion has also been proposed, with high predictive value^21,22^. The spatial sensitivity of electrical functional measures using monopolar cochlear stimulation is, however, limited^23^ and does not reveal which portion of the auditory nerve is responsible for the response. While in animals stimulation is possible both in the primary afferent and the axon, depending on polarity^17^, in many patients the primary afferent is not present and only the central axon is stimulated^24^. However, the response threshold has been suggested to be influenced by the distance of the neural elements from the electrode^25^, provided that hair cells – that interfere by decreasing response thresholds^26–30^—are not present in the cochlea. Thus, if spiral ganglion cells were fully preserved and functional, the threshold should correlate with the modiolar distance of the electrodes. Given that the electric field spreads in all dimensions, threshold measured in current should increase with the square of the distance^31,32^; however, morphology of the stimulated signal and the size, orientation, tissue resistivity and myelination of the stimulated elements play additional roles (see pages 115–134 in^33^). When the current is measured in current level, a logarithmic measure of current, the relationship should be linear. Degenerative changes in spiral ganglion cells are likely to change these relationships substantially.

Consequently, quantifying the relationship between distance to the auditory nerve and threshold may provide an insight into degenerative processes in the spiral ganglion. A precondition is the exact knowledge of the distance of the electrode contacts to the neural elements to be stimulated.

Although the relationship is far from perfect, behavioral threshold can be objectively assessed using recordings of electrically-evoked compound action potentials of the stimulated auditory nerve (eCAP). The present study aims at a systematic analysis of the dependence of compound action potential thresholds on the distance of the electrode from the modiolus. The measurement of intraoperative eCAP-thresholds is part of the daily routine of cochlear implantation to obtain first functional data for programming the speech processor. In this study, we assessed the Euclidean distance of each electrode from the mid-modiolar axis using both intraoperative fluoroscopy and postoperative cone-beam computed tomography (CT). Here, mid-modiolar axis was taken as a proxy of the auditory nerve. We first implanted the cochlea as deep as possible, determined eCAP thresholds, then slightly pulled the electrode back (pull-back technique^34,35^), and the eCAP thresholds were re-measured. By controlling this procedure using intraoperative fluoroscopy, the distances between the electrode contacts and the modiolus could be assessed. Measurements related to mid-modiolar distance, electrode insertion properties, and eCAP thresholds were examined in relation to speech perception. Strong positive correlations were found between threshold and mid-modiolar distance. The slope of these threshold-distance functions significantly correlated with speech perception.

## Materials and methods

This retrospective analysis was based on a review of hospital records from March 2021 to July 2022. Eligible patients were adult, postlingually deaf, native speakers of German who underwent cochlear implantation with a Cochlear™ Nucleus^®^ CI632 implant (Slim Modiolar electrode array) under fluoroscopy. The application of these inclusion criteria resulted in a dataset comprising 15 cochlear implants in 14 patients, aged 38–80 years (mean ± SD: 62.9 ± 13.8 years, see Table 1), representing procedures performed by five different surgeons. One of the patients received two cochlear implants. None of the subjects had significant residual hearing.

Given that all subjects in our center have cochlear lateral wall measurement before implantation^36^, these could be directly retrieved from the database and covered the described range quite well^36^ (Table 1). Similarly, the standardized speech outcomes one year after implantation covered the expected range and included poor and excellent performers (Table 1).

The electrode array used (Slim Midmodialar array) consists of 22 platinum ribbon electrodes in a silicone elastomer, with electrode 22 being the most apical electrode and electrode 1 the most basal electrode. In the curled state, the distance between the centers of the electrode contacts is nominally 0.6 mm, with a contact area between 0.15 mm^2^ and 0.16 mm^2^. When straightened, the electrode array has a length of 14 mm between the most apical and basal electrode.

**Table 1:** Patient demographics obtained from hospital records.

ID	Gender	Age	Duration of loss	Hearing status	Cochlear length	Postoperative WRS	Postoperative OLSA
		[Years]	[Years]	[Loss]	[mm]	[%]	[dB SNR]
P1	W	80	16	Severe	36.83	85	– 2.3
P2	M	71	18	Severe	33.92	90	1.7
P3	M	45	43	Severe	39.33	70	6.4
P4	M	66	50	Profound	37.38	60	0.9
P5	W	54	47	Profound	32.7	45	12
P6	M	74	Unknown	Profound	34.25	50	9.9
P7L	M	66	0	Profound	39.18	75	– 2.8
P7R			0	Profound	39.7	75	– 1.6
P8	M	38	34	Severe	38.8	60	0.8
P9	W	83	Unknown	Profound	36.2	55	– 2.9
P10	M	48	35	Profound	36.6	25	12
P11	M	51	13	Severe	32.7	0	12
P12	W	68	9	Profound	36.2	50	3.3
P13	M	56	Unknown	Severe	38.5	40	
P14	W	80	10	Profound	35	80	2.6

ID – identification number; WRS – word recognition score; OLSA – Oldenburg sentence test; dB SNR – dB signal to noise ratio. Severe hearing loss: 70–90 dB SPL; profound hearing loss: > 90 dB SPL.

We used a standardized clinical procedure to implant the electrodes and evaluated the data retrospectively. All patients gave written informed consent to participate in scientific evaluations and the Ethics Committee of Hannover Medical School has approved the publication of data collected in clinical routine in an anonymized form (ID 1897–2013). All methods were performed in accordance with the relevant guidelines and regulations, including the 1964 Helsinki Declaration and its later amendments.

The insertion of the implant was performed under fluoroscopic control using a medical C-arm (Cios Fusion, Siemens Healthcare) to optimize the electrode placement. Fluoroscopy is a method of continuous visualization of the implantation procedure under continuous X-rays. The fluoroscopy system was aligned to project in the direction of the mid-modiolar axis. This alignment minimized projection errors orthogonal to the mid-modiolar axis and provided a clear view of the electrode array position and the distances of electrode contacts from the modiolus in the resulting fluoroscopy images.

**Fig. 1:**
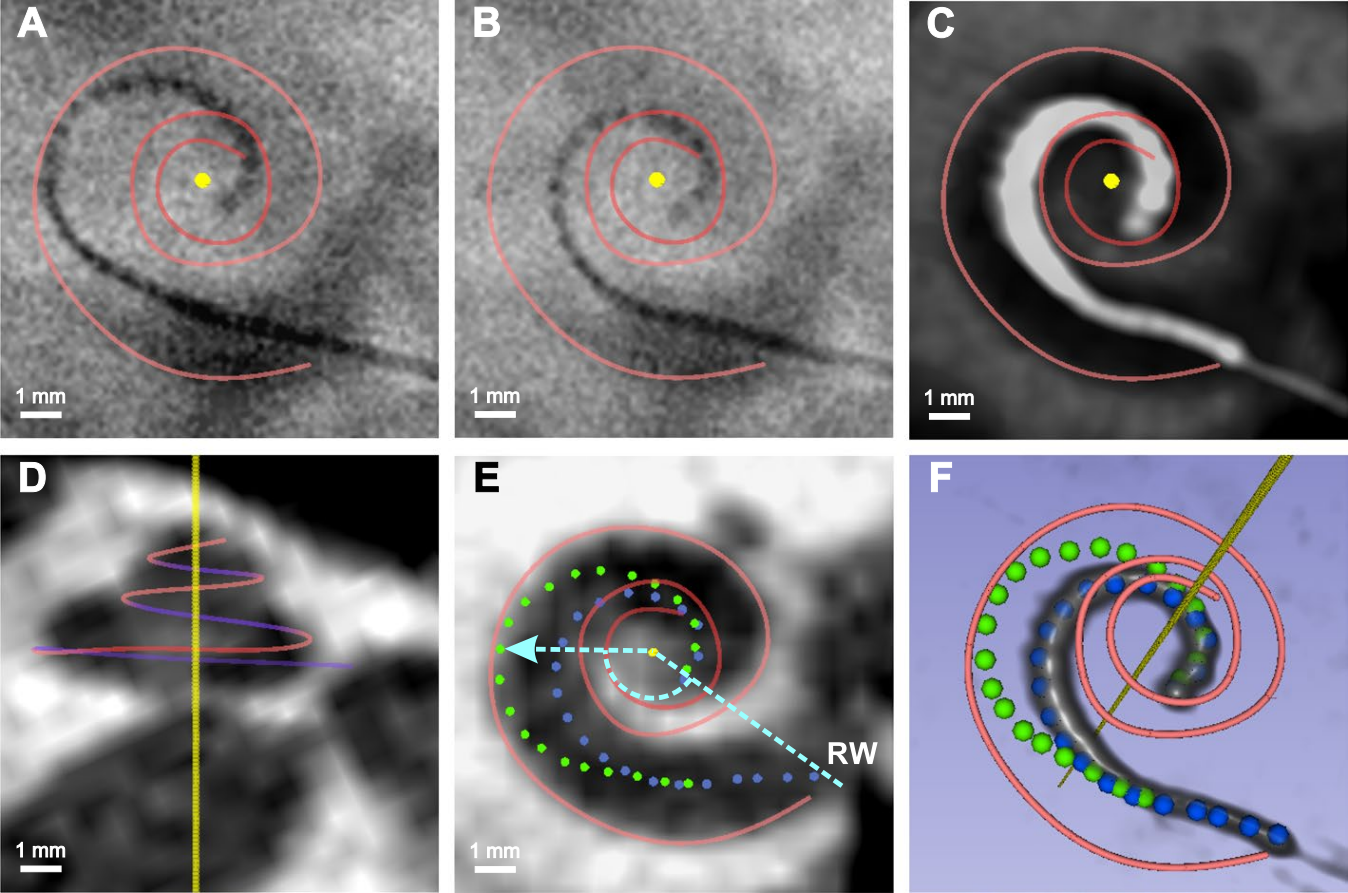
Overview of imaging techniques, registration, and measurements. Example of the fluoroscopy at maximal insertion (**A**) and pull-back (**B**) positions with reconstruction of outer wall spline (red) and mid-modiolar axis (yellow dot). Preoperative (**D**, **E**) and postoperative (**C**) CBCT images with indication of the scala tympani outer wall (red spline) mid-modiolar axis (yellow) and round window (RW). An example measurement of insertion angle and distance is indicated with a dashed cyan line. (**F**) 3D reconstruction of the final electrode position with scala tympani outer wall (red spline), calculated mid-modiolar axis (yellow axis), and individual electrode locations at maximal insertion (green dots) and pull-back (blue dots) positions. The blue markers overlap with the actual position of the cochlear implant.

In the first step, the electrode was advanced under fluoroscopic control until a maximum insertion depth was reached (Fig. 1A). eCAP responses were measured using the AutoNRT function implemented in CustomSoundEP (Version 6.0.201.9). CustomSoundEP provides an intraoperative and a postoperative version for this measurement, of which the intraoperative version with the default parameters was used. Prior to measurement, the electrodes were conditioned with at least two bursts of high current (160 CL, corresponding to 314.73 µA) reducing the impedance of the electrode. The current level was increased using the standard setting of the AutoNRT algorithm by 6 current levels (CLs that are logarithmically related to the current in µA, with 1 CLs corresponding to 0.157 dB current change^37^) until a response was identified on two consecutive measurements. Then stimulation levels were reduced in 2 CL steps until no response was recorded. The eCAP threshold was defined as stimulation level at which a response became just detectable. Subsequently, the current levels were converted to µA according to the manufacturer’s specifications. The detailed algorithm can be found in^38,39^. Stimulation was applied in monopolar configuration and was with charge-balanced biphasic pulses (25 µs/phase) with 7 µs interphase gap. eCAP measurement was performed at a recording electrode that was 2 apical (next-but-one in apical direction, standard procedure), except for the two most apical electrodes, where the recording electrodes were the next-but-one in basal direction.

Then the electrode array was manually slightly retracted under fluoroscopic control (pull-back technique^34,35^). Care was taken to ensure that the tip did not move while the medial region of the implant approached the modiolus. As soon as the tip started to move or the optimal position close to the modiolus was reached, the retraction was stopped (Fig. 1B), and the eCAP thresholds were measured at this final electrode array position. Subsequently, the electrode lead was placed in the mastoid cavity. Care was taken to ensure that the electrode array maintained its pull-back position in the cochlea during electrode lead manipulation.

### Image analysis

For image analysis the consensus cochlear coordinate system^40^ was used. There, the axis of the modiolus coincides with the z dimension. For the analysis of the position of the electrode array in the cochlea, preoperative and postoperative cone beam computed tomography (CBCT) scans (Xoran Technology MiniCAT, 0.3–0.1 mm resolution) as well as intraoperative fluoroscopic radiographs (using a Siemens Healthcare Cios Fusion mobile C-arm) were obtained from the clinical image repository (Fig. 1A–E). These images were analyzed with 3DSlicer^41^ (Version 5.2.1, “http://www.slicer.org”).

In the next step, to determine the mid-modiolar axis, a spline was placed along the outer wall of the cochlea in the preoperative Cone Beam Computed Tomography (CBCT) dataset (Fig. 1, red spline). This has then been used to calculate the axis of rotation corresponding to the mid-modiolar axis (Fig. 1 yellow) following^42^. Pre- and post-operative cone-beam CT volumes were first manually registered using linear rigid registration with the semicircular canals and cochlea as landmarks. Following this initial registration, the BRAINS general registration method was employed^43^ using rigid linear transform and 20% of voxel samples. Individual electrode contacts in the postoperative CBCT volume were marked as fixed landmarks, while corresponding electrode contacts in the final fluoroscopy image were marked as moving landmarks. Fiducial registration was then performed using a similarity transformation, which preserved the fluoroscopy image’s shape while allowing uniform scaling, rotation, and translation to align the electrode array position with the postoperative CBCT.

Fluoroscopy collapses one dimension of the three-dimensional relationships of the implanted cochlea, resulting in projection issues of converting a complex three-dimensional structure into a two-dimensional plane. This is inherent in the method. As one consequence, registration between the postoperative CBCT and the fluoroscopy image may not achieve a perfect match. With the knowledge of this issue, we minimized the error by positioning of the head so that the cochlea was as parallel to the sensor as possible.

The transformation derived from the registration between the postoperative CBCT volume and the final fluoroscopy image was applied to both the final and pre-pull-back fluoroscopy images, thereby registering both fluoroscopy images to the CBCT volumes. Using the registered fluoroscopic images, distance to the mid-modiolar axis and insertion angle of the individual electrodes were determined (Fig. 1E).

### Speech tests

Clinical outcome measures after 1 year of cochlear implant use were obtained from patient records in the clinical database as performed in all subjects. These included the “Freiburger” (German) Monosyllabic Word Test in quiet at 65 dB SPL and the Oldenburg Sentence Test (OLSA) in the S0N0 condition, with fixed noise at 60 dB SPL and an adaptive speech signal converging to a 50% speech reception threshold (SRT). The maximum value for the SRT in noise was set to 12 dB SNR, a limit applied to patients with a higher SRT or when no measurement was possible due to a lack of speech intelligibility in quiet. Both tests were presented from a loudspeaker at a distance of 1 m in front of the subject in a routine clinical room. Contralateral ear was masked with speech-shaped noise if at least one of the frequencies between 250 and 3000 Hz had a threshold better than 30 dB HL or plugged if a threshold was better than 70 dB HL. Any hearing devices were removed from the contralateral ear. For patient P13, who was lost to follow-up care after 3 months for unknown reasons, clinical performance measures from the last available visit were used.

### Data analysis

Statistical analysis and linear fitting procedures were conducted using Matlab (R2022b, MathWorks, Natick, Massachusetts, United States). A two-way repeated measures analysis of variance (rmANOVA) was performed to assess the main effects of condition (maximal insertion vs. after pull-back), electrode contact (positions 1–22), and their interaction on the dependent variable, distance to the mid-modiolar axis.

The rmANOVA was performed using the ranova.m function with both main effects and their interaction included in the model. Post hoc pairwise comparisons between conditions at each electrode were performed using the multcompare.m function. To control for multiple testing across all electrodes, p-values were adjusted using the Bonferroni method. In this procedure, each p-value from the pairwise tests was multiplied by the number of comparisons (22 electrodes). The magnitude of distance change at each electrode was further quantified using Cohen’s d with meanEffectSize.m function.

Independent-sample t-test was conducted in Matlab using the ttest2.m function to compare measurements between two independent groups. Paired-sample t-tests were performed using the ttest.m function when comparing matched measurements from the same subjects under two different conditions. Linear fitting was performed using robust fitting with a bisquare weight function. It was used for determining the slopes for individual subjects as well as for the relationship of the slopes to the speech performance outcomes. Slopes are presented as the estimated value ± 95% confidence interval (CI95). Root mean square error calculation was performed using the Matlab rmse.m function. It is defined as the square root of the sum of the squared difference of the individual measured datapoint from the corresponding fitted regression point. Critical values of correlations coefficients were determined based on the t-distribution for n-2 degress of freedom with two-tailed testing against 0^44^. All significance levels were at α = 5%. Data is provided here as means ± standard deviation.

## Results

**Fig. 2:**
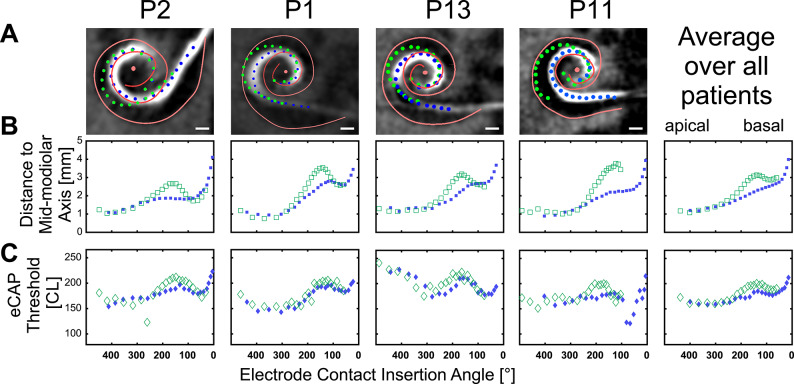
Measurements of representative individual subjects. The four columns display individual patient data for patients P2, P1, P13 and P11. The last column represents the average of all subjects. Row (**A**) displays an overlay of the postoperative CBCT image and the marked positions of individual electrodes at maximal insertion (green circles) and pull-back (blue circles). Row (**B**) illustrates the insertion depth (represented as the insertion angle) and distance from the mid-modiolar axis for individual electrodes. The maximal insertion position and the final pull-back position are shown as green open and blue closed squares, respectively. Row (**C**) reveals the eCAP thresholds of individual electrodes for both positions (open and closed diamonds represent maximal insertion and pull-back positions, respectively).

Four representative examples of registered pre- and postoperative CBCT and the overlayed location of individual electrode contacts identified from fluoroscopy images demonstrate that in each case the maximal insertion (green) shows more abmodiolar locations than the pull-back position (blue, Fig. 2A). We computed the Euclidean distance between the electrode and the closest point of the modiolar axis in 3 dimensions (Fig. 2A, B) for each electrode in each subject in both the maximal insertion and the pull-back position. It revealed that it was mostly 100–250° where the electrodes moved to more modiolar positions after pull back. Interestingly, the most basal electrodes often increased the distance to the mid-modiolar axis after pulled back. This increasing distance is paralleled by a slight increase in eCAP thresholds at these electrodes (Fig. 2C).

**Fig. 3:**
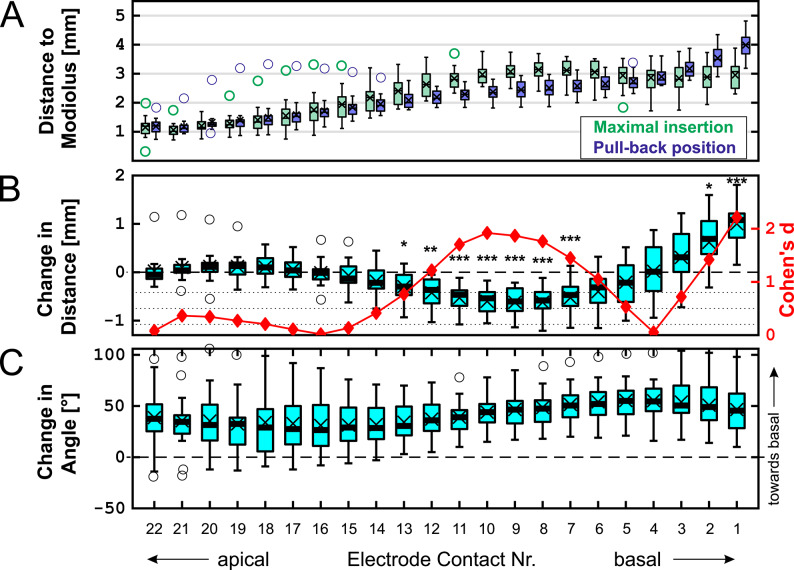
Anatomical changes caused by the pull-back technique. The central line indicates the median, and the box represents the interquartile range (IQR). Whiskers extend to the most extreme values within 1.5 × IQR. Data points beyond this range are shown individually as outliers (circles). The mean is marked with a ‘×’. (**A**) Distances to the modiolus for individual electrodes at both positions. (**B**) Pairwise change in distance after pull-back, calculated as pull-back distance minus maximal insertion distance; negative values indicate movement towards the mid-modiolar axis, with the corresponding values shown on the left axis. A red line with diamond markers shows Cohen’s d for the change, plotted with its own scale on the right axis of the panel. Dotted lines indicate the conventional thresholds for Cohen’s d (0.2, 0.5, and 0.8 for small, medium, and large effects, respectively) Asterisks denote statistically significant differences between conditions at the corresponding electrode positions after Bonferroni-adjusted post hoc pairwise comparisons (*** *p* < 0.001; ** *p* < 0.01; * *p* < 0.05). (**C**) Change in insertion angle in degrees; positive values indicate a basal shift.

We subsequently performed a rmANOVA to examine the effects of condition (maximal insertion vs. after pull-back), electrode contact, and their interaction on distance to the mid-modiolar axis (Fig. 3A, B). The main effect of electrode contact was highly significant (F(21,294) = 84.16, *p* < 0.001), indicating systematic differences in distance along the electrode array. The main effect of condition (maximal insertion vs. after pull-back) did not reach statistical significance (F(1,14) = 4.22, *p* = 0.059), suggesting that, when averaged across all electrodes, pull-back did not result in a significant overall change in distance. Importantly, the interaction between condition and electrode contact was highly significant (F(21,294) = 23.00, *p* < 0.001), indicating that the effect of pull-back on distance to the mid-modiolar axis varied across electrode positions.

Post hoc pairwise comparisons between conditions were conducted separately at each electrode and p-values were Bonferroni-adjusted to control the family-wise error rate across all electrode-wise comparisons. This analysis revealed that pull-back resulted in a significantly decreased distance to the modiolus for electrodes 7 to 13, with electrode 9 moving on average 0.62 ± 0.3 mm towards the modiolus. Two most basal electrodes significantly increased the distance to the modiolus due their movement towards the round window, for electrode contact 1 it was 1.00 ± 0.48 mm.

The magnitude of the distance change induced by pull-back, quantified as Cohen’s d, was largest at the basal end of the array (e.g., up to d = 2.1 at electrode 1), raised again above 0.5 for medial electrode contacts 5 to 13 and diminished toward the apex.

**Fig. 4:**
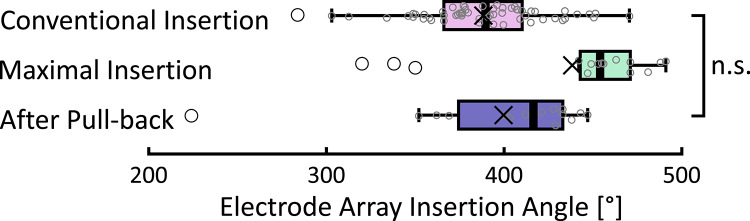
Comparison of electrode array insertion angles at maximal insertion (green) and after pull-back (blue) to conventional insertions (magenta) of the same CI type reported by previous publication. Conventional Insertion of 58 Nucleus CI532/CI632 implants reported previously^45^. Box plots display the median and interquartile range (IQR). Whiskers extend to the most extreme values within 1.5 × IQR, with individual measurements in this range shown as grey circles. Data points beyond this range are shown individually as outliers (black circles). The mean is marked with a ‘×’.

In addition to assessing movement of the electrode contacts towards or away from the modiolus, we also calculated shifts in the angular positions of individual electrode contacts after pull-back. These results are presented in Fig. 3C. One potential disadvantage of the pull-back technique is a reduced insertion angle of the electrode array. Therefore, we compared the insertion angle of the most apical electrode contact (22) in the present study to data from a previous study conducted at our center (see Fig. 4)^45^. In the conventional insertion group, electrode array insertions angles were collected from 58 CI532/CI632 implantations performed at our center prior to the adoption of the pull-back technique. These data serve as a historical reference cohort, allowing direct comparison with final position in the present cohort. The results show a modest change in the angular position of the apical electrode contact after pull-back, with the insertion angle decreasing from 438 ± 55° before to 399 ± 52° after pull-back (Table 1; Fig. 4). This indicates that this electrode shifted toward the angular position of more basal electrodes, to the extent of 39 ± 33° (see change in position of electrode 22 in Fig. 3C) after pull-back. Although the insertion angle is slightly reduced after pull-back, the final insertion angles—both mean and median—are actually improved compared to conventional insertions (average insertion angle of 387 ± 52°) as shown in the reference group. An unpaired t-test revealed no statistically significant difference between the two groups (*p* = 0.35).

To evaluate the overall quality of electrode array placement for each patient, summary measures were used to condense the 22 individual electrode contact measurements into a single, representative value per electrode array. For insertion angle, this was achieved by selecting the most apical electrode contact as the representative electrode array insertion angle. For mid-modiolar distance, the measurements of all 22 electrode contacts after the pull-back manoeuvre were averaged to estimate the final overall distance. For eCAP threshold, all 22 thresholds after pull-back were likewise averaged to yield a single representative threshold value for the electrode array. These three summary measures are presented in Table 2, columns 2, 3, and 4, respectively.

**Fig. 5:**
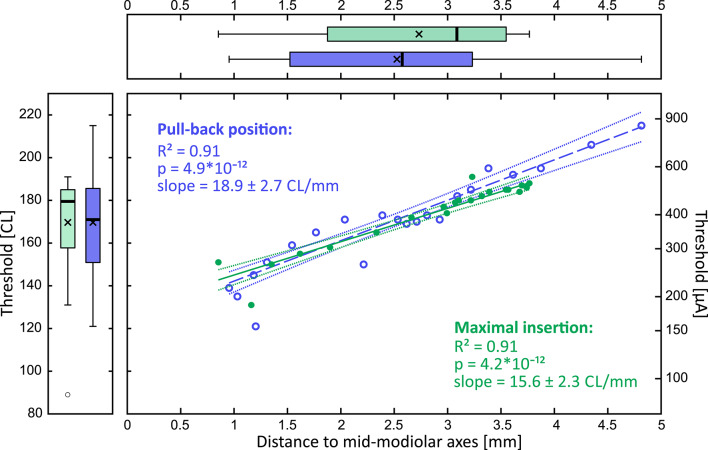
Comparison of the threshold-distance relations for maximal insertion and pull-back position. Shown is subject P3 as example. Green color (closed circles) denotes the maximal insertion, blue (open circles) the pull-back position. The slope, representing the change in neural response threshold per unit change in electrode position, is shown with its 95% confidence interval. The correlations did not change for the two positions, while in the pull-back position some electrodes decreased, but some increased the distance to the modiolus (the basalmost electrodes). Left and top panels show the whisker plots for threshold and distance (i.e. the range of the values). The difference for maximal insertion and pull-back position was not significant (two-tailed t-test, *p* > 0.05).

In the next step, we related the electrode distance to stimulation threshold in each patient to calculate the individual threshold–distance functions. Linear regression was performed using a robust bisquare-weighted fitting approach to minimize the influence of outliers, providing R², p-values, slope estimates, and RMSE for each fit (subject with highest correlation shown in Fig. 5). The results of the regressions are presented in Table 2, columns 5, 6, 7 and 8, respectively. The correlation was significant in all but one subject (mean R^2^: 0.65 ± 0.26).

**Table 2:** Measures of electrode array placement and threshold-distance regression results.

ID	Insertion angle	MMA distance average	eCAP threshold average	Distance vs. threshold		Slope both positions ± CI95	RMSE both positions	Slope final position ± CI95
	[°]	[mm]	[CL]	*R* ^2^	*p*	[CL/mm]	[CL]	[CL/mm]
P1	455	2.2	176.6	0.85	1.20E-18	20.9 ± 2.8	7.8	24.5 ± 3.2
P2	409	2.0	183.4	0.66	1.80E-11	21.7 ± 4.8	10.0	19.6 ± 4.1
P3	365	2.5	169.7	0.90	2.00E-22	17.4 ± 1.8	6.1	18.9 ± 2.7
P4	453	2.0	162.8	0.84	3.50E-18	22.2 ± 3.0	7.0	25.2 ± 4.1
P5	422	2.0	176.0	0.77	4.00E-15	16.6 ± 2.8	8.0	12.7 ± 4.3
P6	409	2.3	177.6	0.65	4.50E-11	17.2 ± 3.9	10.3	15.8 ± 5.8
P7L	409	2.3	167.0	0.76	1.90E-14	22.1 ± 4.0	10.0	20.3 ± 8.3
P7R	378	2.1	163.5	0.64	6.40E-11	21.9 ± 5.1	12.1	21.0 ± 6.9
P8	430	2.1	169.7	0.85	7.40E-19	20.2 ± 2.7	7.2	18.1 ± 4.8
P9	442	2.1	175.5	0.87	5.10E-20	21.8 ± 2.7	7.2	26.0 ± 4.5
P10	334	2.5	181.1	0.59	1.40E-09	11.5 ± 3.0	7.9	12.2 ± 3.9
P11	393	2.1	168.8	0.30	0.00011	8.0 ± 4.1	12.5	9.0 ± 6.9
P12	243	2.8	180.9	0.18	0.004	10.2 ± 6.8	15.6	12.5 ± 12.2
P13	435	2.2	195.9	0.02	0.35	– 3.6 ± 7.7	18.4	– 8.6 ± 10.5
P14	406	2.3	209.0	0.81	1.00E-16	18.0 ± 2.7	8.9	19.9 ± 3.8

“Insertion angle” provides the insertion angle of the most apical electrode contact 22. MMA – mid-modiolar axis. CI95–95% confidence interval. RMSE – root mean square error. “Both positions” refers to pooled data for all electrodes from both maximal insertion and pull-back position.

**Fig. 6:**
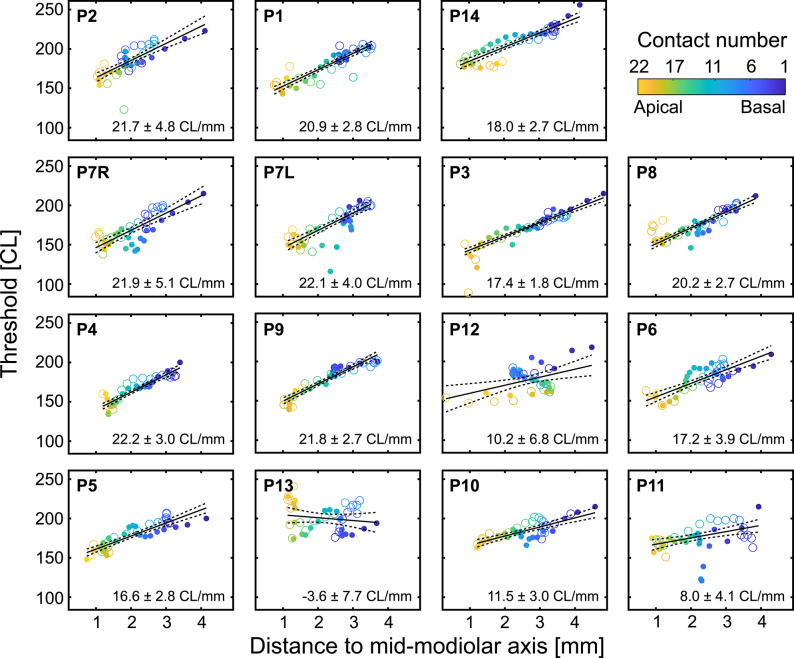
Threshold-distance functions and the corresponding slopes for all subjects in the study. In the 13 of 14 subjects the thresholds significantly correlated with distance (Table 2). The slopes varied from − 3.6 CL/mm to 22.2 CL/mm and are presented with the CI95. Open circles represent the maximum insertion, filled circles represent the pull-back position. Slopes were calculated for the pooled dataset.

We analyzed the slopes of the threshold-distance functions in all subjects (Fig. 6; Table 2). The mean slope of pooled maximal and pull-back position was 16.4 ± 6.9 CL/mm, the mean slope for pull-back position was 16.5 ± 8.3 CL/mm (two-tailed paired t-test, *p* = 0.934).

**Fig. 7:**
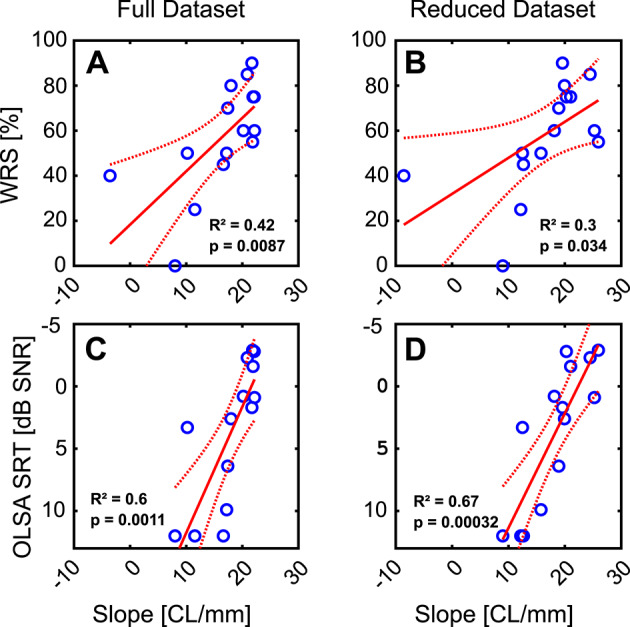
Relation of distance vs. eCAP threshold linear fit slope to speech performance. Shown are outcome of speech intelligibility in quiet (**A**, **B**) and in noise (**C**, **D**). Correlation of individual patient slopes from distance and eCAP thresholds were measured for the pooled 44 measurements (after maximal insertion and final position after pull-back, Full dataset in **A**, **C**), or for the pull-back position only (Reduced dataset, **B**, **D**). Numerical values presented in Tables 1 and 2. One patient without postoperative measurement in noise was excluded from the analyses presented in **C** and **D**.

Given these large differences in slopes between participants, we correlated the measures of electrode array placement and threshold-distance regression results (Table 2) with the German monosyllabic words test and the OLSA sentence test in noise (Fig. 7; Table 3). Whereas insertion angle of the apicalmost electrode, the average distance to the modiolus (from all electrodes), the average eCAP threshold value (from all electrodes) and RMSE error did not significantly correlate with speech outcomes (*p* > 0.05), the slope in both positions and the final pull-back slope of threshold-distance functions correlated significantly (*p* < 0.05).

In many clinics fluoroscopy is not a standard approach and therefore only the data on the final position as obtained in the cone-beam CT may be available. Since this may affect the estimation precision, we analyzed the slopes both the pooled dataset (maximal insertion and pull-back, “Full Dataset”) and the final pull-back position (“Reduced Dataset”). It is relevant that it is only the reduced dataset that in fact influences the performance after implantation, since the pull-back position is the one that the patient uses after the surgery. To obtain knowledge of how the reduced datapoints may affect the outcomes, we also included the “Full Dataset” in the data.

For the full dataset the slope explained 42% (R^2^ = 0.42) of the variability in the monosyllabic word test (Fig. 7A). In the reduced dataset it was still 30% (Fig. 7B). For the sentence test in noise the full dataset the slope explained 60% of the variability of the sentence test in noise (Fig. 7C). In the reduced dataset the variability explained was even 67% (Fig. 7D).

**Table 3:** Correlations of implantation measures with word recognition test and matrix sentence test.

	WRS		OLSA	
	*R* ^2^	*p*	*R* ^2^	*p*
Insertion angle	0.05	0.45	0.08	0.32
Distance	0.02	0.63	0.04	0.49
Threshold	0.04	0.46	0.12	0.23
Slope both positions	**0.42**	**0.0087**	**0.6**	**0.0011**
Slope pull-back only	**0.3**	**0.034**	**0.67**	**0.00032**
RMSE	0.07	0.34	0.02	0.66

Significant p-values of the correlation and corresponding R² values shown in bold. WRS – Freiburg monosyllabic word recognition score. OLSA – Oldenburg matrix sentence test in noise. RMSE – root mean square error.

## Discussion

The presented data confirm that it is the distance to the modiolus — and thus the distance to the neurons stimulated — that extensively impacts the thresholds of electrically evoked compound action potentials in humans. A significant correlation was found in all but one subject, with the portion of variability in thresholds attributable to distance reaching up to 91%. This high explanatory power underscores the importance of precisely controlling the implant’s position in the scala tympani during surgery. Here we employed the pull-back technique^35^ to minimize the electrode–modiolus distance. We demonstrated that for the middle of the electrode array the technique does significantly reduce the distance to the modiolus, whereas for the basalmost electrode it increases it.

The slope of threshold-distance functions explained up to 67% of the variability in speech-in-noise scores in the present study. None of the other factors analyzed had this predictive power (see Table 3). Given the variance in the slopes of threshold-distance function.

### Pull-back technique

The pull-back surgical technique improves thresholds of compound action potentials in patients, reducing the spread of excitation^35^. It was shown effective also for the Slim Modiolar electrode in temporal bones^46^. The technique affected electrical thresholds and speech intelligibility^47^ similarly as in this study. A significant reduction of NRT levels for electrode contacts 6 to 11 as well as a significant increase for electrode 2 and 3 was found, corresponding to 6–14 and 1–3 in the present study. Unfortunately, the previous study^47^ did not quantify the individual electrode-modiolar distances. The effect on speech understanding by the pull-back was very limited with no statistically significant differences in speech intelligibility at 6 and 12 month follow up^47^. Since this was observed in a group comparison, it does not directly imply that the position of the electrodes does not affect performance in the individual patient: in a large cohort of patients, wrapping factor did affect speech reception^7,8^.

We used the pull-back technique in order to minimize the distance of the electrode contacts. We did not see a statistically significant correlation between speech intelligibility and mid-modiolar distance or insertion angle (Table 3). Whereas the Holden studies^7,8^ quantified wrapping factor, the present study quantified individual electrode distances. Furthermore, in the present study a smaller patient group was analyzed, and the group did not include subjects with extreme lateral wall positions and shallow insertion angles. The absence of such unfavorable electrode positions in the present study (that was optimized for a near ideal position) is likely the main reason for the different results, as it reduced outcome variance that might have eliminated the correlation with speech performance.

The electrode array insertion angles of the same CI type reported by previous publications with conventional insertion (387° ± 33° in 58 Nucleus CI532/CI632 implants^45^) were similar to the present study using the pull-back under fluoroscopy (Fig. 4). Real-time visual control of the electrode array with pull-back thus enables minimizing the distance between the electrode contacts and modiolus for each patient without the risk of reducing the insertion angle.

The fluoroscopy includes the disadvantage of higher radiation exposure. At present, there is no alternative to fluoroscopy for checking the position of the electrode in the inner ear live in the operating theater. Thanks to the widespread use of C-arms and their frequent use in operations, it is currently a cost-effective, widely used procedure with a calculable radiation risk for the patient and surgical team^48^. In the future we hope to replace the position control of the electrode in the inner ear with a purely electrical measurement instead of fluoroscopy. A promising technique is transimpedance measurement^49^.

### Electrode distance to the modiolus

Electrode distance to the modiolus explained up to ~ 90% of variability in thresholds in some participants. This observation documents that the distance to the modiolus is a critical factor for thresholds. The threshold to electrical stimulation has been suggested previously to be mainly related to distance from the excitable elements^25,31,32^, with relationship to the volume of the scala tympani^50^ and the resulting current spread^23^. Ideally, neuronal threshold is related to the square of the distance of the electrode to stimulated neural elements^33^. In the present dataset, the threshold indeed linearly correlated with current level. With increasing distance, the amount of excited neural elements at threshold increases due to the higher current needed to reach them, leading to a larger spread of the electrical field. Even monopolar stimulation shows a spatial tuning in auditory nerve fibers for cochleae corresponding in size to humans^23^. Therefore, such effect can be expected at currents close to threshold. There is an additional constraining factor related to the size of the modiolus^51^ relative to the spatial spread of the electrical field that may show up at suprathreshold current levels. Here we only analyzed threshold relations and such suprathreshold effects were therefore not considered.

We propose that the slope of the distance-threshold function reflects the functional state of the spiral ganglion population. Decreasing the distance in a well-preserved population of the spiral ganglion cells should decrease the thresholds more in well-preserved spiral ganglion populations. The slope computed from all electrode contacts would reflect a global measure of the state of the spiral ganglion, of course not sensitive to patchy degeneration that affects the relationship locally. This might be better reflected in RMSE or correlations, but in the present dataset we could not identify a clear effects in these, but individual patients in this dataset might suggest such effect (for example P11 with outliers and a high RMSE, or P13 with high RMSE). Slopes and correlations thus might provide complementary information. However, more studies of these aspects are required to be able to make firm, statistically-backed conclusions. Slopes compared to correlations or RMSE are less dependent on outliers and less dependent on the exact distances (and their count) at which the implant is located.

However, the threshold-distance function is not only dependent on spiral ganglion presence, it is additionally affected by neuronal properties, including the extent of myelination and density of the neurons at the target structure^33^. “Healthier” cochleae tend to have lower electrical stimulation thresholds^52^.

Another element for considering thresholds are electrophonic responses caused by activation of hair cells^26,53^. These reduce the thresholds^29,30^ and increase the dynamic range in mean by 3.4 ± 2.3 dB for apical portions of the cochlea and by 8.9 ± 4.3 dB for basal portion of the cochlea^54^. Electrophonic response are therefore confounding factors of threshold-distance relationships. In the present dataset none of the subjects had a significant residual hearing. Using the here introduced approach electrophonic responses can be safely excluded due to (i) no functional hearing and (ii) use of stimuli that are not effective drivers of electrophonic responses (such as individual pulses with phase durations < 50 µs^30^). While even in absent functional hearing some remaining hair cells may have survived, point (ii) excludes a major influence of electrophony on the present outcomes. Nonetheless, mild electrophonic effects beyond the point of their best cochlear location are still possible^55^.

### Factors affecting neuronal survival

The integrity of the spiral ganglion cells is determined by trophic factors secreted from hair cells and supporting cells^56^. However, in high-frequency regions of the human cochlea the degeneration of spiral ganglion cells does not correlate with hair cell loss^57,58^. Similarly, in congenitally deaf cats the spiral ganglion cells survive over long periods of time despite congenital deafness and complete absence of hair cells^59,60^. This is critically different from neonatal deafening using ototoxic substances with pronounced loss of spiral ganglion cells^61–65^. Neonatal deafening typically results in destruction of all cell material at the basilar membrane, including supporting cells, effectively removing all potential support from the spiral ganglion cells. In natural congenitally deafness, significant cell material is typically preserved in the organ of Corti, despite complete absence of hair cells. Additional to the effect of activity^63^, the supporting cells are thus a key factor in preserving the integrity of spiral ganglion cells in deafness. Therefore, the amount of spiral ganglion degeneration is very likely dependent on the combined extent of degeneration of hair cells and supporting cells, and thus on the etiology of hearing loss.

Consequently, after long period of deafness, human spiral ganglion neurons degenerate and in mean only ~ 30% of the spiral ganglion population survives^66–68^. In the very rare data available from subjects who passed away and donated their temporal bones to research, their speech performance was correlated with the number of surviving spiral ganglion cells^69^, explaining 39% of the variability in CNC word score recognition, but other reports failed to demonstrate such a relationship^70,71^. In the present data, while the ground truth on histology of the spiral ganglion survival was not available, there was a significant positive relationship between the slopes of the threshold-distance function and both word scores and sentence recognition, with the correlation in the case of the reduced data set (the one the subjects wears) and sentence recognition accounting for 67% of the variability. Based on the suspected mechanism responsible for the slopes, we propose the hypothesis that the slope of the threshold-distance function reflects spiral ganglion survival. The high percentage of variability explained suggest that peripheral factors are the main bottleneck for speech performance in postlingual deafness. Pre-implantation electrocochleography can explain 40% or more of the variability in word scores after implantation^21,72^, and similarly, hearing performance before implantation is a predictor of cochlear implant outcome^5^. All this supports that the pre-implantation hearing might correlate with the status of the spiral ganglion. Additional factors involve central adaptation to hearing loss^73^ and higher-order brain factors^6^. The slope of the threshold–distance functions ranged here from − 4 CL/mm (~ − 0.6 dB/mm) to 22 CL/mm (~ 3.45 dB/mm). These slopes were well comparable to previous findings with monopolar stimulation (2 ± 2 dB/mm) and substantially smaller than those observed for the experimental tripolar (13 ± 11 dB/mm) or bipolar (10 ± 8 dB/mm) stimulation^37,74^.

However, the status of the spiral ganglion is typically variable along the cochlea^15,75^. One limitation of the present measure (slope of the threshold-distance function) is that it reflects the overall neural health and does not directly quantify “patchyness” of the degeneration. Thresholds with tripolar stimulation^23^ can provide such information^37,76^, as well as compound action potential recordings with masking stimuli^77^ or polarity effect^78^. The only measure that reached a similar explanatory power as the slope of the threshold-distance functions was obtained with focused stimulation, where CNC word score variance was explained by the error of the threshold-distance model in 63%^37^. In the present study we could explain similar extent of variability using monopolar stimulation, as clinically used in CI subjects.

Direct correlations of eCAP amplitudes and latencies to the spiral ganglion neuron survival in guinea pigs reached 42–60% of variability explained by neuronal degeneration^19^, values similar to speech outcomes in this study. Given that speech is affected by more than just neural degeneration, our approach appears to reflect the degeneration better than amplitudes or latencies of eCAPs. Another suitable measure of neural health may be the slope of interphase-gap functions, as suggested previously^18,79^. These functions have the potential to reflect auditory nerve degeneration and possibly also provide electrode-specific information, uncovering patchy patterns of degeneration. However, it has the disadvantage of requiring dedicated stimulation, at the cost of additional measurement time in patients. The variance of the distance of stimulating electrodes to the modiolus was not assessed in any of the above guinea pig experiments, likely representing a critical biasing factor.

To the best of our knowledge, the amount of variability in speech understanding explained by a single electrophysiological measure reported in the present study is the highest in the literature using the clinical monopolar stimulation. It is likely due to the possibility to assess the distance to the modiolus, a factor that is likely affecting many objective measures of cochlear implant function. Given the same stimulation sensitivity of neurons, increasing the electrode distance to the spiral ganglion increases the current required to achieve the current density required for reaching excitation threshold. At the same time, the sphere defined by this isopotential plane has a larger diameter, thus co-activating more neurons. Very likely this leads to an increase in amplitude of neural responses reflected in compound action potentials. The failure index, defined as a relationship of eCAP saturation amplitude divided by the current required to reach it, has been suggested as an alternative measure that can provide this complementary information on animals^80^. It is interesting that the failure index computationally compensates for the (unknown) distance to the modiolus. The standout advantage of the present study is the possibility to quantify distance. Further quantification of relationships between slope of distance-threshold functions and failure index will be the topic of future studies.

## Conclusion

Distance of electrodes to neural structures is the main determinant of stimulation thresholds in well-preserved neuronal populations. Neuronal degeneration extensively affects this relationship and can be quantified using threshold-distance functions. Provided that distance to the mid-modiolar axis can be assessed, the slope of the threshold-distance function strongly correlates with to speech perception in CI subjects even when stimulated with monopolar configuration. The threshold-distance slope is thus a candidate to measure of neural health.

## Data Availability

The datasets used and analysed during the current study available from the corresponding author on reasonable request.
